# Cervical cancer in women under 30 years of age in Norway: a population-based cohort study

**DOI:** 10.1186/s12905-021-01242-3

**Published:** 2021-03-18

**Authors:** Brit Helene Gravdal, Stefan Lönnberg, Gry Baadstrand Skare, Gerhard Sulo, Tone Bjørge

**Affiliations:** 1 Department of Global Public Health and Primary Care, University of Bergen, 5020 Bergen, Norway; 2 Cancer Registry of Norway, Oslo, Norway; 3 Norwegian Institute of Public Health, Bergen, Norway

**Keywords:** Cervical cancer, Young women, Population-based

## Abstract

**Background:**

We compared women with incident cervical cancer under the age of 30 with older women with regard to stage, morphology, screening history and cervical cancer mortality in a population-based cohort study.

**Methods:**

We included data from the Cancer Registry of Norway. Incidence rates (per 100,000 women-years) were calculated and joinpoint regression was used to analyse trends. The Nelson-Aalen cumulative hazard function for risk of cervical cancer death during a 15-year follow-up was displayed. The hazard ratios (HRs) of cervical cancer mortality with 95% confidence intervals (CIs) were derived from Cox regression models.

**Results:**

The incidence of cervical cancer in women under the age of 30 has almost tripled since the 1950s, with the steepest increase during 1955–80 (with an annual percentage change (APC) of 7.1% (95%CI 4.4–9.8)) and also an increase after 2004 (3.8% (95%CI -1.3–9.2)). Out of 21,160 women with cervical cancer (1953–2013), 5.3% were younger than 30 years. A lower proportion of younger women were diagnosed at more advanced stages and a slightly higher proportion were diagnosed with adenocarcinoma and adenosquamous carcinoma comparing women above 30 years. The cumulative risk of cervical cancer death was lower for patients under the age of 30. However, the difference between the age groups decreased over time. The overall adjusted HR of cervical cancer mortality was 0.69 (95% CI 0.58–0.82) in women diagnosed under the age of 30 compared to older women.

**Conclusion:**

There has been an increase in cervical cancer incidence in women under the age of 30. Cervical cancer in younger women was not more advanced at diagnosis compared to older women, and the cervical cancer mortality was lower.

**Supplementary information:**

The online version contains supplementary material available at 10.1186/s12905-021-01242-3.

## Background

Cervical cancer is the fourth most common cancer in women globally and affects women of all ages [1]. It is the second most common form of cancer in women 15–44 years [2]. A nationwide cervical cancer screening programme was introduced in Norway in 1995 [3]. However, prior to organized screening, there was extensive opportunistic screening [4, 5]. The primary aim of the screening programme is to reduce the incidence and mortality of the disease by identifying and treating its precursor lesions before they develop into cancer [6]. The programme recommends screening every three years for women between 25 and 69 years of age. The screening programme is based on reminders and relies on centralised registration and monitoring of all cervical cytology, human papillomavirus (HPV) tests, cervical histology and treatment of cervical intraepithelial neoplasia (CIN) lesions and cancer [7]. In 2015, the randomised implementation of HPV primary screening in women aged 34–69 commenced in four counties in Norway, replacing cytology [8]. Since 2019, HPV primary screening is gradually being introduced in the remaining Norwegian counties.

In Norway, a reduction of 68% in total cervical cancer incidence due to screening has been estimated [9]. However, there has been an increase in cervical cancer incidence in women under 30 years over the past 20–25 years in Norway and other European countries, such as the UK [7, 10]. The reasons for the increase are unclear but may include changes in sexual behavior and the burden of associated sexually transmitted infections, including HPV [10, 11].

In a study from the UK, cervical cancer in young women (under 25 years) tended to be more aggressive and advanced at the time of diagnosis (stage 1B+ or worse) than if diagnosed in older women (25–29 years) [10]. Furthermore, a higher proportion of younger women were diagnosed with adenosquamous carcinoma and other rarer histological types [10]. Also, the participation rates in cervical cancer screening programmes have slowly decreased in young women in many developed countries in recent years for no clear reason [12].

In this study, we aimed to compare stage, histology, screening history and subsequent cervical cancer mortality in women diagnosed with cervical cancer under 30 years of age (overall and stratified into < 25 and 25–29 years) with those diagnosed with cervical cancer at an older age in Norway.

## Methods

### Data sources

The Cancer Registry of Norway (CRN) was established in 1953 and contains mandatory information on all new cancer cases and precancerous lesions. Information from clinical notifications, pathological notifications and death certificates are the main reporting sources and provide information about site, histological type and stage of disease at the time of diagnosis. The coding and classification system at the CRN is in accordance with international standards [13]. The CRN has also recorded causes of death for cancer patients (from the Cause of Death Registry [14], run by the Norwegian Institute of Public Health), available from 1960.

The Norwegian Cervical Cancer Screening Programme (NCCSP) is an integrated part of the national health care system. The CRN runs the program and receives mandatory reports from private and public pathology and microbiology laboratories. The programme keeps complete records of the results of all pap smears, histology specimens and HPV tests. Individual screening data are recorded and organized into four sub-registries: The Cytology Register, the Histology Register, the HPV Test Register and the CIN Register, the last containing follow-up and treatment data. The SNOMED coding system, with some local adaptations, is used for classification (cytology and histology).

All residents in Norway are assigned a unique identification number used in all administrative and medical registries/databases. This identification number enables accurate record linkage.

### Study population

This cohort study included all women diagnosed with cervical cancer (International Classification of Diseases (ICD)-10; C53) in Norway during 1953–2013 (n = 21,160). No cases with ICD-10 code 55 were included. For 34 women who had two cervical cancer diagnoses, only the first recorded diagnosis was included.

### Statistical analysis

Period of diagnosis (1953–68, 1969–83, 1984–98, and 1999–13), stage distribution (International Federation of Gynecology and Obstetrics: FIGO (1986); stages 1–4), morphology (squamous cell carcinoma, adenocarcinoma, adenosquamous carcinoma, other malignancies and unspecified morphology) [9] and screening history; smears taken from 3.5 years and up to six months prior to diagnosis (no smear, only normal smears, abnormal smears and only unsatisfactory smears), by age groups (< 25, 25–29 and ≥ 30 years) were descriptively displayed and analysed using contingency tables. Information on screening history was available for cancers diagnosed from 1 July, 1995 onwards [15]. Chi-square tests were used to evaluate differences in distributions between age groups. A p-value below 0.05 was considered statistically significant.

**Fig. 1:**
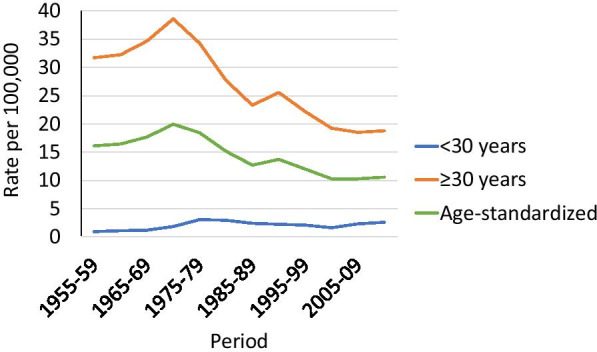
Crude incidence rates (per 100,000 women-years) by age (< 30 and ≥ 30 years) and age-standardised rates (World standard population) of cervical cancer, Norway, 1955–2014

Crude incidence rates (per 100,000 women-years) of cervical cancer by age group (< 30 and ≥ 30 years) and age-standardised rates (World standard population), 1955–2014, were calculated separately using supporting data from the CRN (Fig. 1), taking immigration and emigration into account. The temporal trends in age-standardised rates were estimated using Joinpoint Trend Analysis Software from the National Cancer Institute (Version 4.8.0.1). We analysed time trends in incidence rates in women < 30 and ≥ 30 years. For the joinpoint analysis we used age-specific numbers of cervical cancer provided by the CRN, population numbers from Statistics Norway, in 12 five-year periods (1955–59, …, 2010–14) and weights for age-standardisation to the World standard population from the WHO [16]. The whole period 1955–2014 was segmented by the points with trend change, and the annual percentage change (APC) in rates between the trend-change points was estimated. Thereafter, the average annual percent change (AAPC) for the whole study period was calculated as a weighted average of the estimated APC in each segment by using the segment lengths as weights [17].

The Nelson-Aalen cumulative hazard function for risk of death from cervical cancer, with 95% confidence intervals (CIs), during a 15-year follow-up by age (< 30 and ≥ 30 years) and period of diagnosis (1960–73, 1974–86, 1987–99 and 2000–13), was calculated [18, 19]. The start of follow-up was from date of diagnosis and the individuals were followed up until emigration, death, 15 years after diagnosis or end of follow-up on 31 December, 2016, whichever occurred first. Follow-up ended 15 years after diagnosis due to the relatively low number of deaths.

Hazard ratios (HRs) of cervical cancer mortality with 95% CIs, with and without adjustment for stage (FIGO stages 1–4), morphology (squamous cell carcinoma, adenocarcinoma, adenosquamous carcinoma, other malignancies and unspecified morphology) and time of diagnosis (continuous), were derived from Cox proportional hazard regression models. We also presented estimates for specific periods of diagnosis (1960–73, 1974–86, 1987–99 and 2000–13).

The data were analysed using IBM SPSS Statistics 22 (IBM Corporation, Armonk, NY, USA) and Stata/IC 14.0 (StataCorp LP, College Station, TX, USA).

## Results

Since the 1950s, the incidence of cervical cancer in women aged < 30 increased from 0.9 per 100,000 women-years in 1955–59 to 2.6 in 2010–14) (Fig. 1). However, the increase has not been steady. The joinpoint regression analysis identified two joinpoints, in 1980 and 2005. After a peak in the 1970s (APC in incidence was 7.1% (95% CI 4.4–9.8) during 1955–80), the incidence slightly decreased in 1980–2005 (APC in incidence was -2% (95%CI -3.5–0.0)) and then increased slightly again after 2004 (APC in incidence was 3.8% (95% CI -1.3–9.2) during 2005–14). For the whole period 1955–2014, the AAPC in incidence was 2.3% (9% CI 1.2–3.4). In contrast, in women aged ≥ 30, the incidence was 31.7 in 1955–59 and declined after a peak in the early 1970s to 18.8 in 2010–14.

The main analysis included a total of 21,160 women diagnosed with cervical cancer in Norway during 1953–2013. Table 1 summarises the characteristics of these women by age at diagnosis. Most women were above 30 years (94.7%). Cervical cancer was rare in women under 25 years of age, with only 207 cases (0.98%) in this age group. In women under 30 years, 87.0% in the age group < 25 years and 82.4% in the age group 25–29 years were diagnosed at stage 1, whereas in women above 30 years, 51.8% were diagnosed at stage 1.

**Table 1:** Characteristics of women diagnosed with cervical cancer in Norway by age, 1953–2013

	All ages		Age < 25		Age 25–29		Age ≥ 30		p-difference^a^
	n	%	n	%	n	%	n	%	
*Period of diagnosis*									
1953–68	5,495	26.0	13	6.3	106	11.6	5,376	26.8	
1969–83	6,168	29.1	85	41.1	263	28.7	5,820	29.0	
1984–98	5,089	24.1	59	28.5	290	31.7	4,740	23.7	
1999–13	4,408	20.8	50	24.2	256	28.0	4,102	20.5	< 0.001
*Stage (FIGO)*									
1	11,322	53.5	180	87.0	754	82.4	10,388	51.8	
2	5,236	24.7	19	9.2	103	11.3	5,114	25.5	
3	2,775	13.1	3	1.4	33	3.6	2,739	13.7	
4	1,487	7.0	3	1.4	9	1.0	1,475	7.4	
Unknown	340	1.6	2	1.0	16	1.7	322	1.6	< 0.001
*Morphology*									
Squamous cell carcinoma	17,565	83.0	164	79.2	745	81.4	16,656	83.1	
Adenocarcinoma	2,284	10.8	26	12.6	104	11.4	2,154	10.7	
Adenosquamous carcinoma	374	1.8	6	2.9	34	3.7	334	1.7	
Other malignancies	880	4.2	10	4.8	28	3.1	842	4.2	
Unspecified morphology	57	0.3	1	0.5	4	0.4	52	0.3	< 0.001
Total	21,160	100.0	207	100.0	915	100.0	20,038	100.0	
*Screening history*^b^									
No smear	3,371	60.3	24	38.7	119	36.8	3,228	62.0	
Only normal smears	1,445	25.9	25	40.3	119	36.8	1,301	25.0	
Abnormal smears^c^	694	12.4	13	21.0	76	23.5	605	11.6	
Only unsatisfactory smears	78	1.4	0	0	9	2.8	69	1.3	< 0.001
Total	5,588	100.0	62	100.0	323	100.0	5,203	100.0	

^a^p-values for difference: Chi-square tests between age groups

^b^Smears taken from 3.5 years and up to six months prior to diagnosis, available from 1 July, 1995

^c^Cytology diagnosis of atypical squamous cells of undetermined significance or more severe

In all cases, the proportion of women diagnosed with stage 1 cancer increased from 42.4% in 1953–68 to 58.8% in 1999–2013. The corresponding proportions in women < 30 years were 59.7% and 81.4%, respectively (Table 2). The majority of women in all age groups were diagnosed with squamous cell carcinoma, but a slightly higher proportion of women under 30 years were diagnosed with adenocarcinoma and adenosquamous carcinoma compared to women above 30 years (Table 1). For women with adenocarcinoma and adenosquamous cell carcinoma, a higher proportion were diagnosed at stage 1 (Table 3) than for women with squamous cell carcinoma. For women under 30 years with adenocarcinoma and adenosquamous carcinoma, 83.1 and 77.5%, respectively, were diagnosed at stage 1.

**Table 2:** FIGO stage by age and period of diagnosis (%) for women diagnosed with cervical cancer in Norway, 1953–2013

Age at diagnosis	Period of diagnosis					
	Stage	1953–68	1969–83	1984–98	1999–2013	Total
< 30 years		(n = 119)	(n = 348)	(n = 349)	(n = 306)	(n = 1,122)
	1	59.7	88.5	87.7	81.4	83.2
	2+	38.7	10.1	10.9	16.7	15.2
	Unknown	1.7	1.4	1.4	2.0	1.6
≥ 30 years		(n = 5,376)	(n = 5,820)	(n = 4,740)	(n = 4,102)	(n = 20,038)
	1	42.0	53.4	56.5	57.1	51.8
	2+	56.2	45.3	42.6	40.2	46.6
	Unknown	1.8	1.3	0.8	2.7	1.6

**Table 3:** FIGO stage by age at diagnosis and morphology (%) for women diagnosed with cervical cancer in Norway, 1953–2013

Age at diagnosis	Stage	Morphology					
		Squamous cell carcinoma	Adenocarcinoma	Adenosquamous carcinoma	Other malignancies	Unspecified morphology	Total
< 30 years		(n = 909)	(n = 130)	(n = 40)	(n = 38)	(n = 5)	(n = 1,122)
	1	84.5	83.1	77.5	63.2	60.0	83.2
	2+	14.6	13.1	22.5	28.9	0.0	15.2
	Unknown	0.9	3.8	0.0	7.9	40.0	1.6
≥ 30 years		(n = 16,656)	(n = 2,154)	(n = 334)	(n = 842)	(n = 52)	(n = 20,038)
	1	51.2	61.5	56.6	39.9	21.2	51.8
	2+	47.5	36,4	42.8	53.0	69.2	46.6
	Unknown	1.3	2.1	0.6	7.1	9.6	1.6

We only had data on screening history available for those women diagnosed from 1 July, 1995 when the national screening programme started. Overall, 60.3% of these women did not have smears taken during the 3.5-year period preceding diagnosis; the corresponding proportion was 62.0% in women above 30 years, 38.7% in women < 25 years and 36.8% in women 25–29 years (Table 1). Also, a higher proportion of women in the younger age group had only normal smears before diagnosis (age < 25; 40.3% and age 25–29; 36.8%), when compared to women above 30 years of age (25.0%). In women under 30 years of age, 21.0% of women under 25 years and 23.5% of women 25–29 years had abnormal smears 3.5 years before diagnosis; 11.6% in women above 30 years. Additional file 1: Table 1 (Table S1) displays similar figures for women in the target age range of the screening programme (25–69 years).

**Fig. 2:**
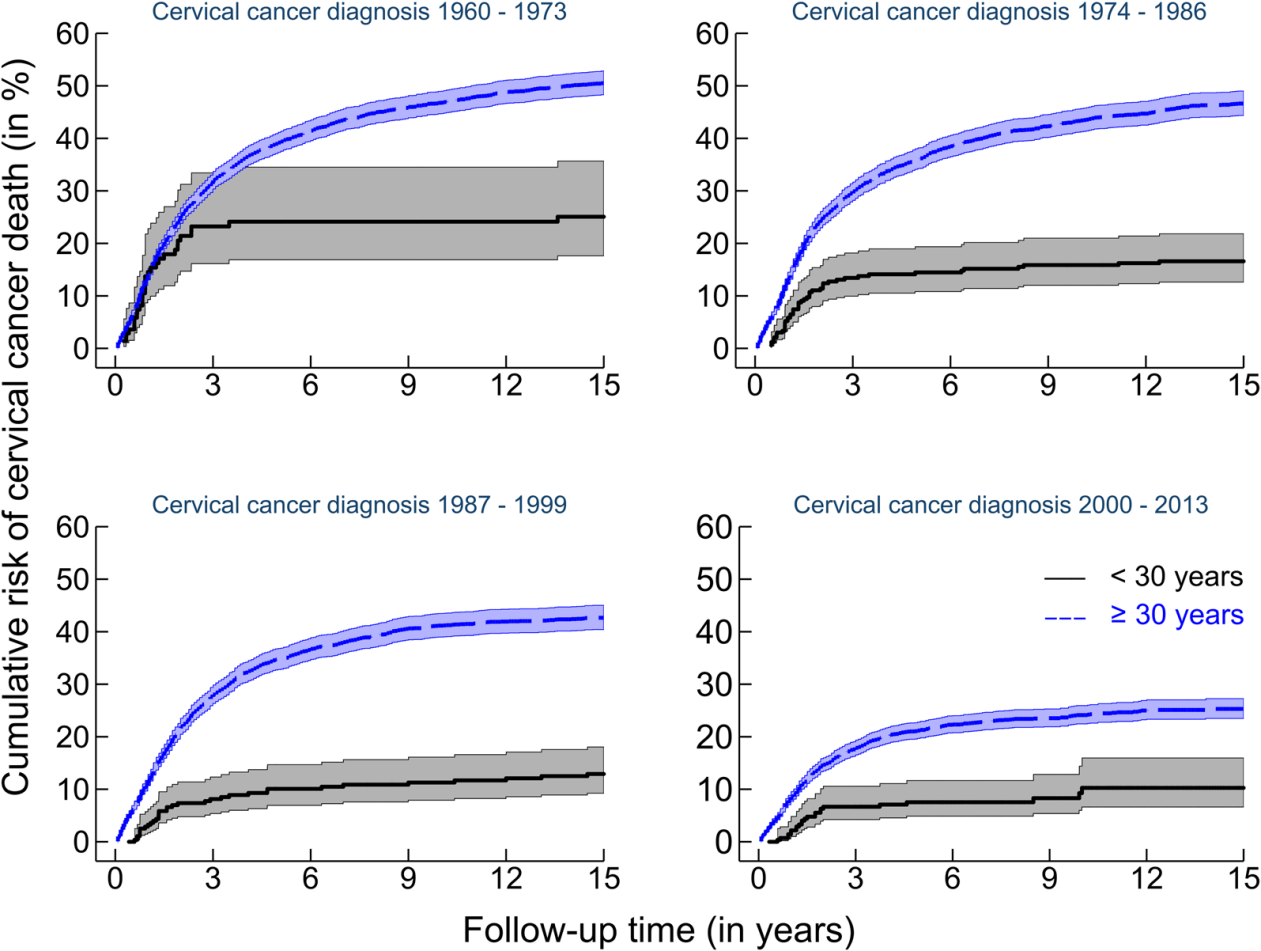
Cumulative risk of death* from cervical cancer^#^, with 95% confidence intervals, during 15 years of follow-up by age (< 30 and ≥ 30 years) and period of diagnosis (1960–73, 1974–86, 1987–99 and 2000–13). * Nelson-Aalen cumulative hazard function, unadjusted estimates [18, 19]. ^#^ 5,980 cervical cancer deaths

Figure 2 shows the cumulative risk of death from cervical cancer during a 15-year follow-up by age (< 30 and ≥ 30 years) and period of diagnosis (1960–73, 1974–86, 1987–99 and 2000–13). The cumulative risk of death from cervical cancer decreased with calendar time for women both under and above 30 years of age. However, the difference between the age groups decreased. The cumulative risk of death from cervical cancer after 15 years of follow-up was higher for women ≥ 30 years than for women < 30 years in all four time periods that were studied.

In the Cox regression analysis of cervical cancer mortality (1960–2013), the women were followed for an average of 8.7 years (range; 0–15 years), constituting 163,500 women-years. During follow-up, 5,980 cervical cancer deaths were identified. The overall unadjusted and adjusted HRs of cervical cancer mortality were 0.38 (95%CI 0.32–0.45) and 0.69 (95% CI 0.58–0.82), respectively, in women diagnosed under the age of 30 compared to older women (Additional file 2: Table S2). The HRs (adjusted for stage and morphology) for the diagnosis periods 1960–73, 1974–86, 1987–99 and 2000–13 were 0.78 (95%CI 0.55–1.13), 0.74 (95%CI 0.56–0.97), 0.56 (95%CI 0.40–0.80), and 0.66 (95%CI 0.43–1.01), respectively.

## Discussion

The incidence of cervical cancer in women under 30 years of age has almost tripled since the 1950s, while decreasing overall. Nevertheless, only a small proportion of women diagnosed with cervical cancer were under 30 years of age. In our study, cancers in younger women were of earlier stage, and only a slightly higher proportion of women had adenocarcinoma and adenosquamous carcinoma compared to women above 30 years of age. Mortality from cervical cancer was lower for women under the age of 30 even after adjusting for stage. The screening history of women with cervical cancer under the age of 30 differed from those older at the time of diagnosis. A larger proportion of the younger women with cancer had preceding screening tests, both normal and abnormal. This may reflect poorer effectiveness of screening in younger age groups, as has been suggested by a number of past publications [20–22].

One of the strengths of this study was its population-based design, including all women with cervical cancer in Norway since the 1950s. Our data set did not include information on hysterectomy. Due to the missing information on hysterectomy, incidence rates particularly among women above 30 years, are likely underestimated as the rates of hysterectomy increase with age. However, the rates of hysterectomy are relatively low in Norway compared to other Western countries [23–25].

Another limitation of the study was the relatively low number of cervical cancer cases in women in the youngest age groups. Also, when examining the screening history in women above the age of 25, we were unable to separate the cytology tests following an invitation to screening from those taken in response to symptoms. In the NCCSP, women are invited to be screened from the age of 25. However, the results of all smears (in all age groups) are recorded. Since women under 25 years of age were not part of the national screening programme for the entire period, self-selection for screening may be more pronounced in this age group and could be influenced by factors such as the use of contraception and associated visits to health care providers, the healthy screenee effect, as well as symptoms.

The cervical cancer screening programme from 1995 with written invitations for pap smears every third year increased coverage of the target population [26]. For women 25–39 years of age there was a decrease in coverage until 2012. However, from 2012 onwards, increased coverage has been noted, particularly in women aged 25–34 years. This could be attributable to media coverage and projects aimed at increasing screening attendance, such as the "Sjekk deg-kampanjen" ran by the Norwegian Cancer Society [7].

A study from Canada published in 2001 [27] concluded that the incidence of invasive cervical adenocarcinoma and adenosquamous carcinoma had been steadily increasing in women 20–49 years of age. Another study from the UK published in 2011 showed that the incidence of cervical cancer in women 20–29 years of age increased significantly after 1992 [11]. On the contrary, a US study (2017) concluded that the incidence of invasive cervical cancer in younger women aged 21–25 was very low and declined significantly between 2000 and 2013 [28]. However, among 24–25-year-olds the incidence remained constant. Our study showed that the cervical cancer rate in women under the age of 30 has almost tripled since the 1950s. However, the incidence of cervical cancer in women both above and under 30 years peaked in the 1970s when opportunistic screening became widespread, with a further decline after the screening programme started. Overall, the proportion of women diagnosed at stage 1 increased over time, and the proportion also increased in women diagnosed under 30 years of age.

A study from the UK published in 2013 concluded that cervical cancer in young women was rare and that only a small group of women 20–29 years of age had been diagnosed with cervical cancer before the age of 25 [10]. Similarly, in our data, only around 1% of the cancers were diagnosed before the age of 25. Like the UK study, our study also showed that a clear majority of women diagnosed at < 30 years were diagnosed at stage 1. The same study from the UK concluded that cervical cancer in young women (aged 20–24 years) tended to be more advanced and was often of a rarer histological type than cancers in older women [10]. In our study, a slightly higher proportion of women in the younger age groups (under 30 years) were diagnosed with either adenocarcinoma or adenosquamous carcinoma, but the cancers in general did not tend to be more advanced. Also, for younger women with adenocarcinoma or adenosquamous carcinoma the majority were diagnosed at stage 1.

A Canadian study from 2012 showed the link between invasive cervical cancer and mortality in young women 15–29 years of age. The study concluded that both the disease and mortality among these women were rare, and had declined during the study period (1970–2007) [29]. Also, an earlier Hungarian study showed no differences in survival among the different age groups, and concluded that cervical cancer in young women was not more aggressive than in other age groups [30]. A further study from England (2012) found that 91% of younger women with cervical cancer were diagnosed at stage 1A or 1B and had an excellent prognosis [31]. All these studies support the results of our study, showing a lower cervical cancer mortality for younger women, when compared to older women. The absolute difference in the cumulative risk of death from cervical cancer between the two age groups in the current study decreased with calendar time, particularly in the periods since 1987. This is probably due to the much stronger impact of the organised screening programme on both incidence and mortality in the age groups above 30 years.

There are still varying opinions regarding the appropriate age to start cervical screening. European guidelines recommend that screening should start from 25–30 years [32]. Australia’s programme starts from age 18 [33], whereas the screening programmes in Finland and The Netherlands start at the age of 30 [34]. Studies have concluded that screening below the age of 25 leads to significant over-treatment with an uncertain impact on cervical cancer incidence and mortality [33, 35]. A study from the UK also concluded that the increase in cervical cancer in young women cannot be attributed to the lack of screening of women aged 20–24 years [31].

In 2009, HPV vaccination was introduced in the Norwegian national vaccination programme for girls in primary school and also included boys from autumn 2018 [36]. It is too early to quantify the long-term effects of the vaccination in Norway, but it is expected that the incidence of cervical cancer in vaccinated women will decrease rapidly. Both vaccines used in Norway have demonstrated good efficacy against the high-risk HPV types 16 and 18, responsible for an estimated 73% of cervical cancer cases in Europe [37, 38]. HPV vaccination will likely affect the incidence of cervical cancer overall due to herd immunity, particularly given the high vaccination coverage in Norway.

## Conclusions

Even though only a relatively small number of cervical cancers occurs in women under 30 years of age, rates in this age group have almost tripled in Norway since the 1950s. Cancer in young women does not tend to be more advanced at diagnosis, compared to older women. Also, the mortality from cervical cancer appears to be lower for women in the younger age groups. While the effectiveness of screening may be age-dependent, hopes are raised for an eventual decrease in cervical cancer burden across all age groups due to HPV immunisation through the Norwegian national vaccination programme from 2009 for all girls born after 1996.

## Supplementary Information

**Additional file 1.Table S1.** Characteristics of women aged 25-69 years diagnosed with cervical cancer in Norway by age, 1953-2013.

**Additional file 2.** **Table S2.** Hazard ratios (HRs) of cervical cancer mortality (with 95% CIs) overall and by period of diagnosis, with and without adjustment for stage and morphology.

## Data Availability

The datasets analysed during the current study are not freely available due to national regulations.

## Abbreviations

CI: Confidence interval
CIN: Cervical intraepithelial neoplasia
CRN: Cancer Registry of Norway
FIGO: International Federation of Gynecology and Obstetrics
HPV: Human papillomavirus
HR: Hazard ratio
ICD: International Classification of Diseases
NCCSP: Norwegian Cervical Cancer Screening Programme
