# Supplementary material for: Cervical cancer in women under 30 years of age in Norway: a population-based cohort study
Source: BMC Womens Health. 2021 Mar 18;21:110. doi: 10.1186/s12905-021-01242-3 (PMC7977265; doi:10.1186/s12905-021-01242-3)
Supplement: Supplementary file 1 — Additional file 1.Table S1. Characteristics of women aged 25-69 years diagnosed with cervical cancer in Norway by age, 1953-2013. [file 12905_2021_1242_MOESM1_ESM.docx]

**Table S1** Characteristics of women aged 25-69 years diagnosed with cervical cancer in Norway by age, 1953-2013

|  | Age 25-69 | | Age 25-29 | | Age 30-69 | | p-difference^1^ |
| --- | --- | --- | --- | --- | --- | --- | --- |
|  | n | % | n | % | n | % |  |
| **Period of diagnosis** |  |  |  |  |  |  |  |
| 1953-68 | 4986 | 27.8 | 106 | 11.6 | 4880 | 28.6 |  |
| 1969-83 | 5276 | 29.4 | 263 | 28.7 | 5013 | 29.4 |  |
| 1984-98 | 4053 | 22.6 | 290 | 31.7 | 3763 | 22.1 |  |
| 1999-13 | 3640 | 20.3 | 256 | 28.0 | 3384 | 19.9 | <0.001 |
| **Stage (FIGO)** |  |  |  |  |  |  |  |
| 1 | 10303 | 57.4 | 754 | 82.4 | 9549 | 56.0 |  |
| 2 | 4358 | 24.3 | 103 | 11.3 | 4255 | 25.0 |  |
| 3 | 2046 | 11.4 | 33 | 3.6 | 2013 | 11.8 |  |
| 4 | 999 | 5.6 | 9 | 1.0 | 990 | 5.8 |  |
| Unknown | 249 | 1.4 | 16 | 1.7 | 233 | 1.4 | <0.001 |
|  |  |  |  |  |  |  |  |
| **Morphology** |  |  |  |  |  |  |  |
| Squamous cell carcinoma | 15014 | 83.6 | 745 | 81.4 | 14269 | 83.7 |  |
| Adenocarcinoma | 1892 | 10.5 | 104 | 11.4 | 1788 | 10.5 |  |
| Adenosquamous carcinoma | 331 | 1.8 | 34 | 3.7 | 297 | 1.7 |  |
| Other malignancies | 679 | 3.8 | 28 | 3.1 | 651 | 3.8 |  |
| Unspecified morphology | 39 | 0.2 | 4 | 0.4 | 35 | 0.2 | <0.001 |
|  |  |  |  |  |  |  |  |
| **Total** | 17955 | 100.0 | 915 | 100.0 | 17040 | 100.0 |  |
|  |  |  |  |  |  |  |  |
|  |  |  |  |  |  |  |  |
| **Screening history**^2^ |  |  |  |  |  |  |  |
| No smear | 2506 | 54.7 | 119 | 36.8 | 2387 | 56.1 |  |
| Only normal smears | 1344 | 29.3 | 119 | 36.8 | 1225 | 28.8 |  |
| Abnormal smears^3^ | 656 | 14.3 | 76 | 23.5 | 580 | 13.6 |  |
| Only unsatisfactory smears | 74 | 1.6 | 9 | 2.8 | 65 | 1.5 | <0.001 |
| Total | 4580 | 100.0 | 323 | 100.0 | 4257 | 100.0 |  |

^1^ p-values for difference: Chi-square tests between age groups

^2^ Smears taken from 3.5 years and up to six months prior to diagnosis, available from 1 July, 1995

^3^ Cytology diagnosis of atypical squamous cells of undetermined significance or more severe
