# Supplementary material for: Cervical cancer in women under 30 years of age in Norway: a population-based cohort study
Source: BMC Womens Health. 2021 Mar 18;21:110. doi: 10.1186/s12905-021-01242-3 (PMC7977265; doi:10.1186/s12905-021-01242-3)
Supplement: Supplementary file 2 — Additional file 2. Table S2. Hazard ratios (HRs) of cervical cancer mortality (with 95% CIs) overall and by period of diagnosis, with and without adjustment for stage and morphology. [file 12905_2021_1242_MOESM2_ESM.docx]

**Table S2** Hazard ratios (HRs) of cervical cancer mortality (with 95% CIs) overall and by period of diagnosis, with and without adjustment for stage and morphology

| Period of diagnosis | Crude HR | 95% CI | Adjusted HR | 95% CI |
| --- | --- | --- | --- | --- |
|  |  |  |  |  |
| 1960-1973 | 0.52 | 0.36-0.74 | 0.78 | 0.55-1.13 |
| 1974-1986 | 0.37 | 0.29-0.48 | 0.74 | 0.56-0.97 |
| 1987-1999 | 0.30 | 0.21-0.42 | 0.56 | 0.40-0.80 |
| 2000-2013 | 0.37 | 0.24-0.56 | 0.66 | 0.43-1.01 |
| Overall | 0.38 | 0.32-0.45 | 0.69* | 0.58-0.82 |

* Additionally adjusted for time of diagnosis (continuous)

Slik det er nå:

|  | Ujustert |  |  |  | Justert |  |  |
| --- | --- | --- | --- | --- | --- | --- | --- |
| Samlet | 0.38 | 0.32 | 0.44 |  | 0.67 | 0.57 | 0.79 |
|  |  |  |  |  |  |  |  |
| 1960-1973 | 0.53 | 0.37 | 0.76 |  | 0.79 | 0.55 | 1.13 |
| 1974-1986 | 0.37 | 0.28 | 0.48 |  | 0.73 | 0.56 | 0.96 |
| 1987-1999 | 0.30 | 0.21 | 0.42 |  | 0.57 | 0.40 | 0.80 |
| 2000-2013 | 0.37 | 0.25 | 0.56 |  | 0.68 | 0.44 | 1.04 |

Korrigert

|  | Ujustert |  |  |  | Justert |  |  |
| --- | --- | --- | --- | --- | --- | --- | --- |
| Samlet | 0.38 | 0.32 | 0.45 |  | 0.69 | 0.58 | 0.82 |
|  |  |  |  |  |  |  |  |
| 1960-1973 | 0.53 | 0.37 | 0.76 |  | 0.79 | 0.55 | 1.13 |
| 1974-1986 | 0.37 | 0.28 | 0.48 |  | 0.73 | 0.56 | 0.96 |
| 1987-1999 | 0.30 | 0.21 | 0.42 |  | 0.57 | 0.40 | 0.80 |
| 2000-2013 | 0.37 | 0.25 | 0.56 |  | 0.68 | 0.44 | 1.04 |

Justert for diagnoseår (enkeltår) stadium og histologi

Ikke justert for diagnoseår (enkeltår)

|  | Ujustert |  |  |  | Justert |  |  |
| --- | --- | --- | --- | --- | --- | --- | --- |
| Samlet | 0.38 | 0.32 | 0.45 |  | 0.65 | 0.55 | 0.77 |
|  |  |  |  |  |  |  |  |
| 1960-1973 | 0.52 | 0.36 | 0.74 |  | 0.78 | 0.55 | 1.13 |
| 1974-1986 | 0.37 | 0.29 | 0.48 |  | 0.74 | 0.56 | 0.97 |
| 1987-1999 | 0.30 | 0.21 | 0.42 |  | 0.56 | 0.40 | 0.80 |
| 2000-2013 | 0.37 | 0.24 | 0.56 |  | 0.66 | 0.43 | 1.01 |
|  |  |  |  |  |  |  |  |
| Justert for stadium og histologi | | |  |  |  |  |  |
